# Post-Stroke Adaptation of Lateral Foot Placement Coordination in Variable Environments

**DOI:** 10.1109/TNSRE.2021.3072252

**Published:** 2021-04-20

**Authors:** Andrew C. Dragunas, Tara Cornwell, Roberto López-Rosado, Keith E. Gordon

**Affiliations:** Department of Physical Therapy and Human Movement Sciences, Northwestern University, Chicago, IL 60611 USA, and also with the Department of Biomedical Engineering, Northwestern University, Chicago, IL 60611 USA; Department of Physical Therapy and Human Movement Sciences, Northwestern University, Chicago, IL 60611 USA; Department of Physical Therapy and Human Movement Sciences, Northwestern University, Chicago, IL 60611 USA, and also with Edward Hines, Jr. VA Hospital, Hines, IL 60141 USA

**Keywords:** Gait, stroke, stability, foot placement

## Abstract

Individuals with stroke often have difficulty modulating their lateral foot placement during gait, a primary strategy for maintaining lateral stability. Our purpose was to understand how individuals with and without stroke adapt their lateral foot placement when walking in an environment that alters center of mass (COM) dynamics and the mechanical requirement to maintain lateral stability. The treadmill walking environments included: 1) a Null Field – where no forces were applied, and 2) a Damping Field – where external forces opposed lateral COM velocity. To evaluate the response to the changes in environment, we quantified the correlation between lateral COM state and lateral foot placement (FP), as well as step width mean and variability. We hypothesized the Damping Field would produce a stabilizing effect and reduce both the COM-FP correlation strength and step width compared to the Null Field. We also hypothesized that individuals with stroke would have a significantly weaker COM-FP correlation than individuals without stroke. Surprisingly, we found no differences in COM-FP correlations between the Damping and Null Fields. We also found that compared to individuals without stroke in the Null Field, individuals with stroke had weaker COM-FP correlations (Paretic < Control: p = 0.001, Non-Paretic < Control: p = 0.007) and wider step widths (p = 0.001). Our results suggest that there is a post-stroke shift towards a non-specific lateral stabilization strategy that relies on wide steps that are less correlated to COM dynamics than in individuals without stroke.

## I. Introduction

PEOPLE with an intact neuro-musculoskeletal system exhibit step-to-step changes to mediolateral foot placement in response to small changes to their center of mass (COM) state (position and velocity) during walking [1]. For example, if the COM is positioned more laterally during the swing phase, people often respond with a wider step. This step-to-step coordination aids in the maintenance of frontal plane stability. Specifically, coordinated modulation of lateral foot placement will scale the medially-directed gravitational moment created about the ankle joint to the immediate demands imposed by the COM state [2]. During the single-limb support phase, active adjustment of lateral foot placement combined with the passive dynamics of the swing limb leads to high correlations between lateral COM state and impending lateral foot placement. By mid-stance, over 80% of the variance in step-to-step lateral foot placement can be explained by lateral COM state, which approaches 100% by initial contact as dictated by passive dynamics [1], [3]–[5]. Collectively, this research suggests that coordinated changes in lateral foot placement resulting from an interplay of both active control strategies and passive dynamics may be an important mechanism for maintaining mediolateral gait stability.

However, for individuals recovering from stroke, the capacity to make coordinated adjustments to lateral foot placement in response to changes in COM state may be impaired. Indeed, recent work by Stimpson *et al.* found evidence that within individuals with stroke, this correlation is weaker for paretic steps than for non-paretic steps [6]. For this population, challenges in coordinating lateral foot placement may be a result of both sensory-motor deficits and changes in lower-body mechanical properties (e.g. joint stiffness). Individuals with stroke exhibit deficits in lateral foot placement control [7], greater variability when prescribed a particular step width [8], and proprioception deficits in muscles that act in the frontal plane [9], each of which may impair the ability to actively coordinate lateral foot placement and COM state. Neurologic changes following a stroke, such as abnormal torque synergies [10], [11] and spasticity [12], alter the coordination of the paretic limb. These neurologic changes affect the available range of motion, force generation, and an individual’s ability to independently control degrees-of-freedom in the lower extremity of both the paretic and non-paretic extremities. In addition, because the lower extremity is mechanically linked to the pelvis, passive transfer of energy across body segments (e.g. from the pelvis to the thigh segment) can also contribute substantially to the coordination between lateral foot placement and COM state [1], [3], [5]. As such, neurologic changes following a stroke may impose limits on this transfer of energy between segments. Together, these factors suggest that the coordinated variability in lateral foot placement in response to fluctuations in COM state will be impaired in post-stroke populations compared to their counterparts without stroke.

The level of step-to-step coordination between lateral COM state and lateral foot placement responds to the continuous demands to maintain lateral stability. Specifically, when the demands to maintain lateral stability are reduced by external lateral forces that resist lateral COM excursions [13], [14] or velocity [15], individuals exhibit decreases in step width and step width variability [13]–[16], as well as a weaker correlation between lateral COM state and lateral foot placement [4]. Conversely, when individuals walk in environments that increase the demands to maintain lateral stability (e.g. a movement amplification force field that amplifies lateral COM velocity), individuals exhibit increases in step width and step width variability [17], [18], as well as a stronger correlation between lateral COM state and lateral foot placement [18].

It is unknown if the correlation between lateral COM state and lateral foot placement will also adapt to the altered demands to maintain lateral stability in individuals with stroke. A recent study found that some individuals with stroke reduced their step width when walking with external lateral stabilization, while others either increased or did not change their step width [19]. The post-stroke coordination of lateral COM state and lateral foot placement has not been examined while walking with external stabilization, but the inconsistent findings in step width changes suggest that the impaired neuromuscular system is limited in its ability to adapt foot placement in response to significant changes in lateral stability demands.

Therefore, our purpose was to better understand how adaptable lateral foot placement is to changes in COM state for individuals with and without a stroke. To accomplish this, individuals with and without stroke walked at their preferred speed during normal steady-state walking (Null Field) and in a velocity-based Damping Field that resists lateral COM velocity and reduces the demands to maintain lateral stabilization. It has been recently shown in neurologically-intact individuals that external fields which limit COM excursion weaken the correlation between COM state and lateral foot placement [4]. Although a velocity-based field is mechanically different from a position-based field in the application of lateral forces, past research suggests that lateral foot placement variability will be affected by manipulation of COM position and velocity [1], [4], [18]. In an environment that reduces the stabilization requirements, fewer or less precise adjustments in compensatory lateral foot placement may be necessary. Therefore, we hypothesized that compared to the Null Field, the correlation between lateral COM state and lateral foot placement would be weaker in the Damping Field across both populations. In addition, we expected the Damping Field to lead to reductions in step width and step width variability compared to the Null Field. We also hypothesized that individuals with stroke would exhibit a weaker correlation between lateral COM state and lateral foot placement across fields compared to their peers without stroke.

## II. Methods

### A. Participants

We collected data and analyzed data from a total of 18 participants. The participants included nine individuals with chronic stroke (7 male, 59 +/−7 years old) and nine individuals without stroke (9 male, 61 +/−6 years old). Both Northwestern University and Edward Hines Jr. Veterans Administration Hospital Institutional Review Boards approved the study protocol and procedures. All participants provided written, informed consent prior to data collection.

Data for individuals without stroke were collected as a part of a separate study [20] that, as described below, used a similar experimental protocol but included small differences in inclusion/exclusion criteria and data collection methods. Individuals with stroke were included if they experienced a stroke at least one year prior to data collection, had unilateral paresis, were not using medications that affect walking ability or balance, and had the ability to walk continuously for 6 minutes without the use of an assistive device. Individuals without stroke were included if they were able to walk 10 minutes without undue fatigue, were not using medications that affect walking ability or balance, and did not have any musculoskeletal or neurological pathology that would affect their walking ability or balance.

General demographic information (all participants) and clinical outcome measures characterizing walking speed (Self-Selected and Fast 10-Meter Walk Test [21]), postural balance (Berg Balance Scale [22]), and lower limb function (Lower Extremity Fugl-Meyer Assessment [23]) for participants with stroke are included in Table I.

### B. Experimental Setup

Participants performed a series of walking trials on an oversized treadmill (2.6 m long × 1.4 m wide) (Tuff Tread, Willis, TX). Participants wore a trunk harness attached to a passive overhead support system that did not provide bodyweight support (Aretech, Ashburn, VA).

During select walking trials, a Damping Field applied external lateral forces to the pelvis. The Damping Field was produced using a cable-driven robotic device, the Agility Trainer, which uses the excursion of the linear motors to estimate COM state [17]. Participants walking in the Damping Field experienced a continuous, lateral force proportional in magnitude and opposite in direction of their real-time lateral COM velocity. The gain for the Damping Field was 50 Ns/m, which was selected as previous research has found that this gain was sufficient to produce significant changes in lateral foot placement [16].

To measure gait kinematics, we used a 12-camera motion capture system (Qualisys, Gothenburg, Sweden) to record the 3D positions of 33 retro-reflective markers at 100 Hz. Markers were affixed bilaterally to the anterior and posterior iliac spines, greater trochanters, lateral epicondyle of the knee, lateral malleoli, calcanei, and the 2nd and 5th metatarsals. Additional 4-marker tracking clusters were affixed bilaterally to the participant’s thigh and shank, and a single marker was placed on the sternum.

### C. Protocol

Prior to walking trials, a licensed physical therapist performed clinical outcome measures to assess walking and balance function for participants with stroke. These clinical assessments included the Lower Extremity Fugl-Meyer Assessment (LE-FMA) [23], Berg Balance Scale (BBS) [22], and the 10 Meter Walk Test (10MWT) [21] at both self-selected and fast speeds. Clinical outcome measures for individuals without stroke included only the 10MWT.

Next, participants with stroke performed walking trials to determine their preferred treadmill walking speed. Participants were asked to subjectively compare gradual increases and decreases in speed until they reached a steady-state speed they felt most comfortable walking at on the treadmill.

Finally, each participant with stroke performed a randomized series of walking trials. Individuals walked at their preferred walking speed with two force field conditions: 1) Null – no applied forces, and 2) Damping – the velocity-based damping field described above was applied. Participants with stroke repeated each condition twice. During each trial, participants walked for a total of 5 minutes: 3 minutes to familiarize and remove any learning effects from previous trials, immediately followed by 2 minutes of walking, during which data were collected. Participants did not use handrails or assistive devices during the walking trials; however, if participants normally wore an ankle-foot orthosis for community ambulation, they were allowed to wear it during the trials (n = 5).

Individuals without stroke performed treadmill walking trials at their preferred speed in Null and Damping Fields using similar protocols to those described above for individuals with stroke. Individuals without stroke walked for 200 continuous steps each trial and performed additional treadmill walking trials that were not analyzed for the current study [20].

### D. Data Analysis

We analyzed walking data collected from individuals with and without stroke. To make comparisons between trials and groups, we chose to analyze only the last 50 steps from each trial. This ensured that any measures that were sensitive to the number of steps analyzed were consistent across all participants.

Kinematic data were processed with Qualisys Track Manager (Qualisys, Gothenburg, Sweden), Visual3D (C-Motion, Germantown, MD), and custom MATLAB (Mathworks, Natick, MA) scripts. Marker data were low-pass filtered (4th-order Butterworth, 6 Hz cut-off frequency) and gap-filled in Visual3D. Gait events (foot-off and initial contact) were identified based on the vertical position of the markers on the calcaneus and 5th metatarsal. Timing of gait events was visually checked for accuracy. Additionally, COM position and velocity were calculated in Visual3D using a pelvis segment that was defined by bilateral markers on the iliac crests and greater trochanters. This estimate of COM state has been shown to minimize bias in estimating COM dynamics [24].

To investigate stepping strategy, a regression equation was used to predict the next mediolateral foot placement based on the COM state (position, relative to the stance foot, and velocity) at discrete time points during the preceding swing phase [1]. This method, described by Wang & Srinivasan, calculates the coefficient of determination (R^2^) as the ratio between the variance in the dependent variable (lateral foot placement position) that is predicted by the independent variables (lateral COM position and velocity) [1]. As the dimensionless unit R^2^ increases, it indicates that there is a smaller difference between the predicted and actual foot placement. Therefore, a greater R^2^ indicates a stronger correlation between COM state and foot placement. To model this stepping strategy, we adapted the following regression equation:

$$FP_{x}=\beta_{1}(i)\cdot COM_{x}(i)+\beta_{2}(i)\cdot COM_{\upsilon }(i)+\varepsilon (i)$$

where *FP_x_* is lateral foot placement position relative to the contralateral stance foot, *COM_x_* is the lateral COM position, *COM*_*v*_ is the lateral COM velocity, *β*_1_ and *β*_2_ are regression coefficients, *ε* is the error, and *i* is a discrete time point during the preceding swing phase. The independent variables (COM state) were demeaned prior to their use in the regression equation. This regression was repeated for every 2% of the preceding swing phase. The resulting time-series quantification of the R^2^ values between the predicted and actual lateral foot placement positions was used as the primary outcome measure. For participants with stroke, separate R^2^ time-series were computed for the paretic and non-paretic extremities, and a single R^2^ time-series was computed for the control extremities. For nomenclature, a paretic R^2^ time-series refers to steps where the paretic extremity is in the swing phase.

We also calculated two additional gait metrics that could provide insight into the strategies used by participants to maintain frontal plane stability. These gait metrics included step width mean and variability. Step width was calculated as the mediolateral distance between the calcaneus markers at initial contact. For individuals with stroke, this metric was separated into a paretic and non-paretic step width (i.e., paretic step width was the lateral distance from the calcaneus of the paretic extremity at initial contact to the position of the non-paretic calcaneus at the preceding initial contact). Step width variability was calculated as the standard deviation of step width.

### E. Statistical Analysis

To compare stepping strategies, we used MATLAB to perform a two-way ANOVA using a statistical parametric mapping (SPM) approach [4]. For a given dataset, the SPM approach regards the data as a vector field which changes in time or space and uses principles from random field theory to calculate the probability that changes in the vector field are due to chance fluctuations [25]. This approach can compare features over the entire time-series and has been shown to be generalizable to a variety of 1−, 2−, and 3-dimensional biomechanical datasets [26], [27]. We performed a two-way ANOVA with fixed effects of limb (Paretic, Non-Paretic, and Control), field (Null and Damping), and the interaction between limb and field. The output of SPM produces a F-value for each sample of the R^2^ time-series. If the F-value for a given time point crosses a set threshold value, corresponding to *α* = 0.05, it indicates a statistically significant effect. If there was a significant main effect of limb or an interaction of limb and field, Bonferroni-corrected pairwise comparisons were made to determine significant differences.

To compare gait metrics, we used SPSS (IBM, Armonk, NY) to create separate linear mixed effects models for the two variables: step width mean and variability. Fixed effects included limb (Paretic, Non-Paretic, and Control), field (Null and Damping), and the interaction between limb and field. Random intercepts allowed each participant to deviate from the main intercept. For participants without stroke, a paired t-test was performed to determine if there was a significant effect from limb side; otherwise, data for the group were collapsed across limbs. If there was a significant interaction between limb and field, Bonferroni-corrected pairwise comparisons were made to determine the significant pair(s) of fields (Null vs. Damping) within each limb and of limbs (Paretic vs. Non-Paretic, Paretic vs. Control, and/or Non-Paretic vs. Control) within each field. Significance was set to p<0.05 for all tests.

## III. Results

### A. Participant Demographics

The nine participants with stroke were 59 ± 7 years old and 7 males/2 females with a preferred treadmill walking speed of 0.43 ± 0.17 m/s. Gait speeds (as indicated by 10-Meter Walk Test) suggest that participants with stroke would be classified as limited community ambulators (gait speed 0.4–0.8 m/s [28]). Scores from the Berg Balance Scale indicate that participants were not at increased risk for falling (Berg > 44 [29]). Scores from Lower Extremity Fugl-Meyer suggest that participants were moderately impaired (LE-FM between 19 and 27, [30]). The nine participants without stroke were 61 ± 6 years old and all males with a preferred treadmill walking speed of 1.04 ± 0.19 m/s. Participant demographics and clinical outcome measures for both groups are included in Table II.

### B. Damping Field

**Fig. 1.**
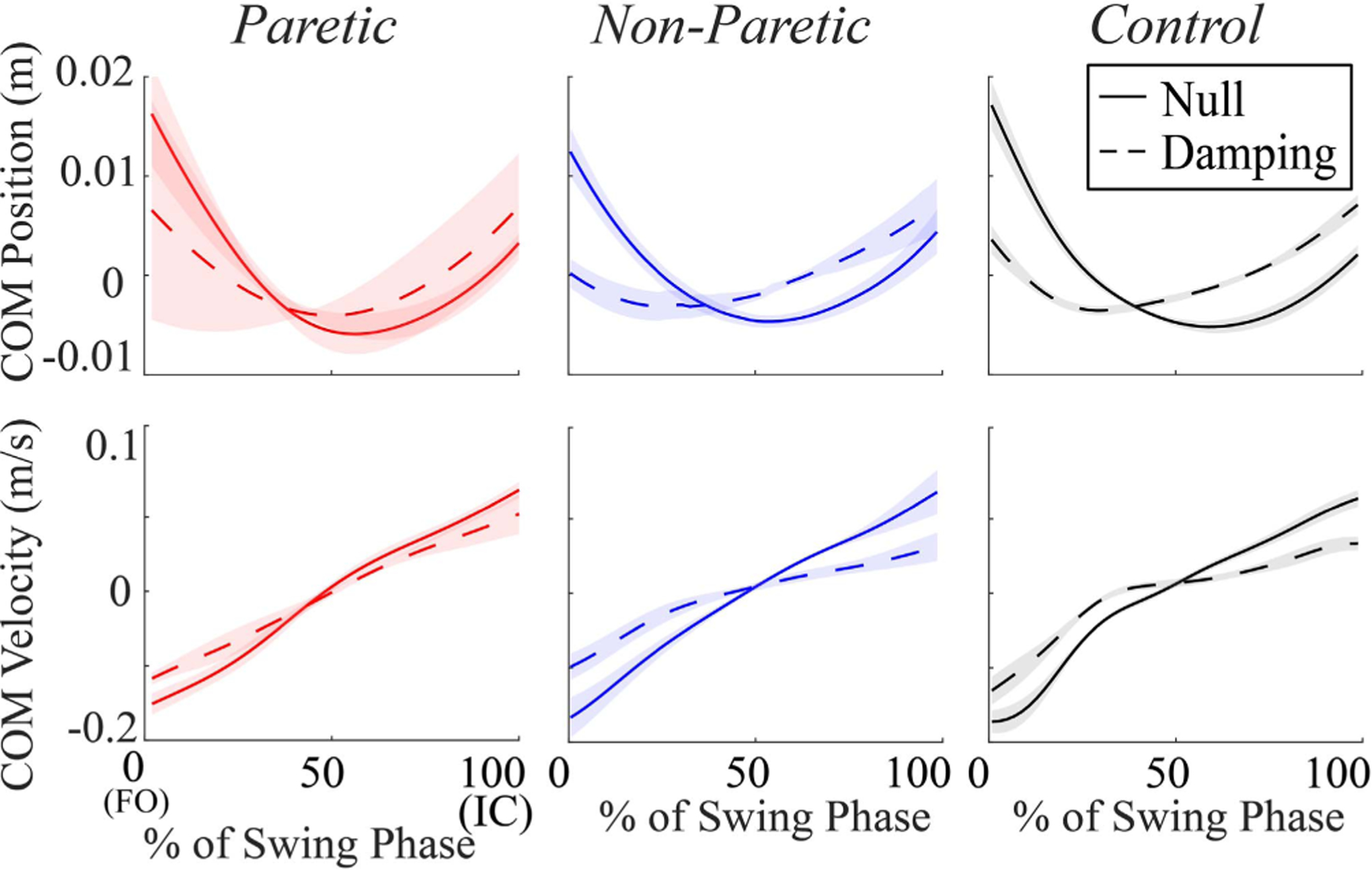
Mean ± standard error of lateral COM position and velocity during swing phase for each limb (Paretic, Non-Paretic, and Control) and field (Null: solid, Damping: dashed).

Lateral COM velocity throughout the swing phase was reduced in the Damping Field in comparison to the Null Field (Fig. 1). The differences in velocity between fields were greatest at foot-off and initial contact. For the participants without stroke, lateral COM velocity decreased by 24% at foot-off and 46% at initial contact in the Damping Field compared to the Null Field. Similar reductions were seen in the participants with stroke during the swing phase of the non-paretic extremity, where COM velocity decreased by 41% at foot-off and 54% at initial contact in the Damping Field compared to the Null Field. During the swing phase of the paretic extremity, lateral COM velocity decreased by 24% for both foot-off and initial contact in the Damping Field compared to the Null Field.

The Damping Field also affected lateral COM position, primarily by creating a change in the relative time when maximum COM excursion occurred during the swing phase. Across all groups, maximum lateral COM excursion during the swing phase was earlier in the Damping Field than in the Null Field. For the participants without stroke, peak COM excursion occurred at 60% of the swing phase in the Null Field but at 30% of the swing phase in the Damping Field. A similar shift occurred during the swing phase of the non-paretic extremity, shifting the time-point of peak COM excursion from 54% of the swing phase in the Null Field to 34% in the Damping Field. A smaller shift occurred during the swing phase of the paretic extremity, where the time-point of peak COM excursion shifted from 56% of the swing phase in the Null Field to 50% in the Damping Field.

### C. Stepping Strategy

**Fig. 2.**
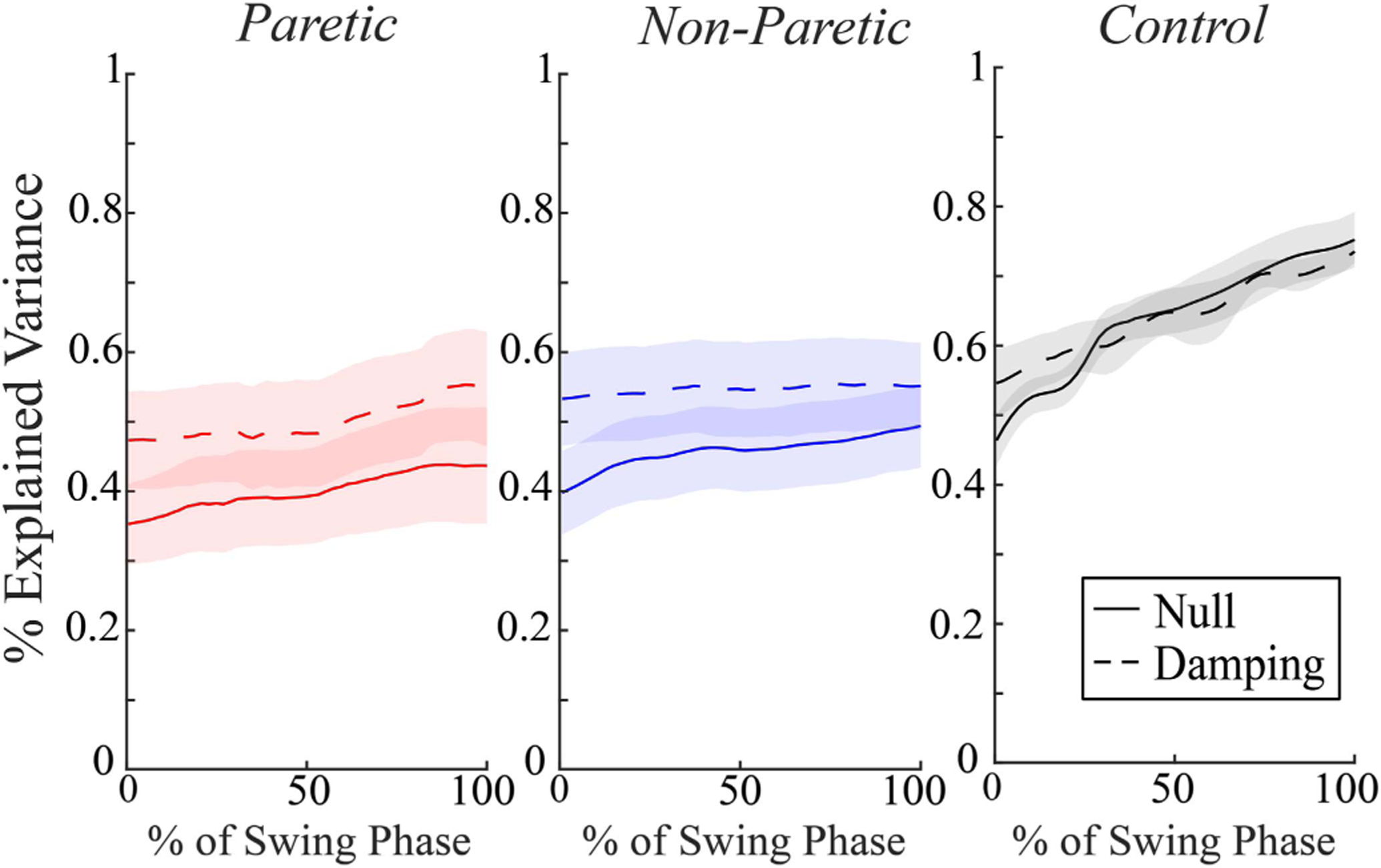
Mean ± standard error (shaded region) of *R*^2^ time-series showing capacity of COM state (position and velocity) to predict subsequent lateral foot placement for limbs (Paretic, Non-Paretic, and Control) and fields (Null: solid, Damping: dashed), throughout the swing phase of gait.

To determine how participants adapted their stepping strategy between limbs and within the fields, we used a regression equation to predict lateral foot placement position based on the lateral COM state (position and velocity) during the preceding swing phase of gait (Fig. 2). This regression equation produced a R^2^ time-series that represents the amount of the variance in foot placement that can be explained by COM state. For all limbs, the R^2^ time-series values tended to increase throughout the preceding swing phase of gait. R^2^ values at the beginning and end of the swing phase are included in Table II.

**Fig. 3. .**
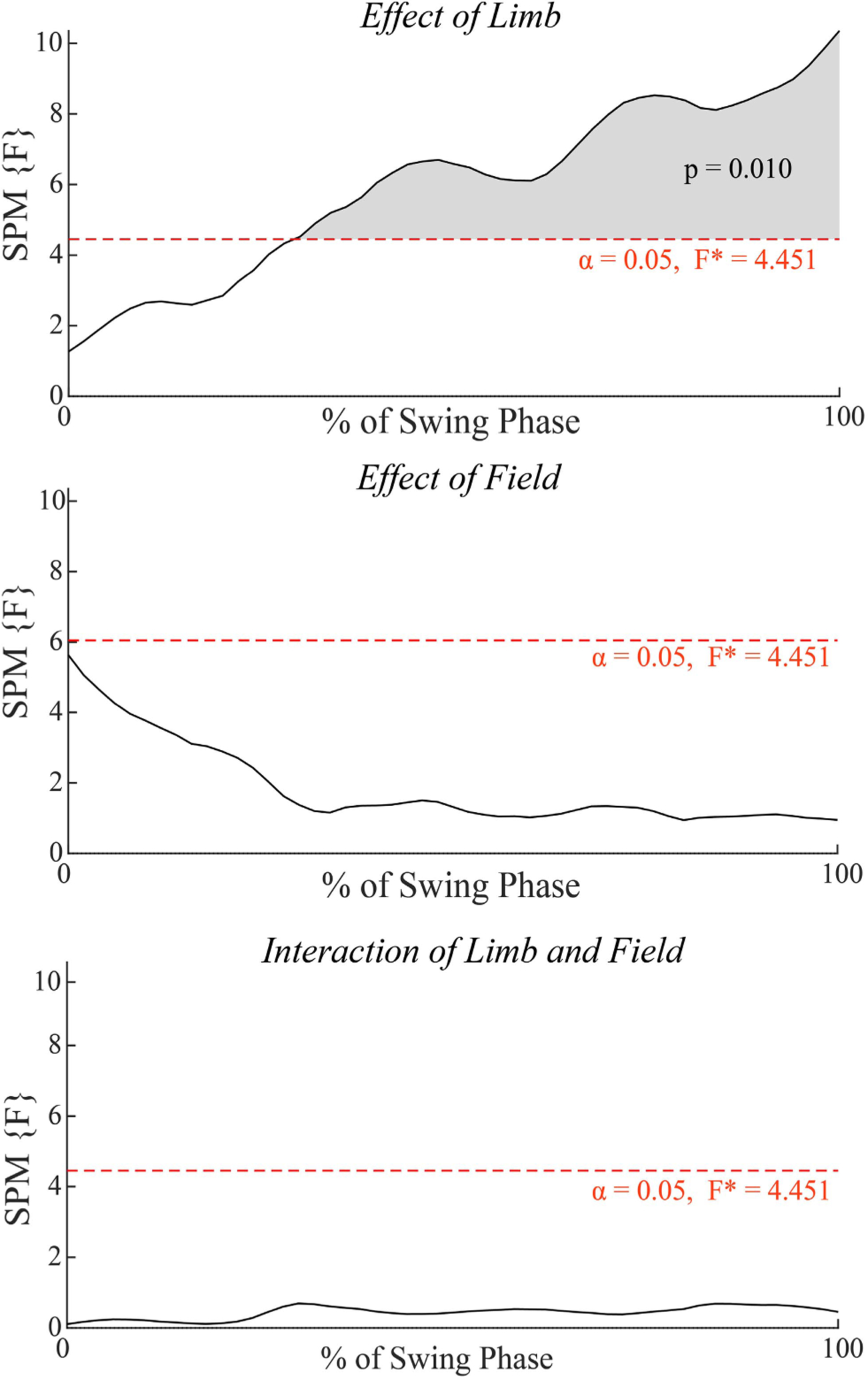
2-way ANOVA with main effects of limb (Paretic, Non-Paretic, Control) and field (Null, Damping) and the interaction of limb and field on R2 time-series. The dashed red line indicates the threshold F-value, corresponding to an < 1 = 0.05, where values above this threshold are statistically significant. Shaded regions indicate significant effects for the corresponding portion of the swing phase.

**Fig. 4.**
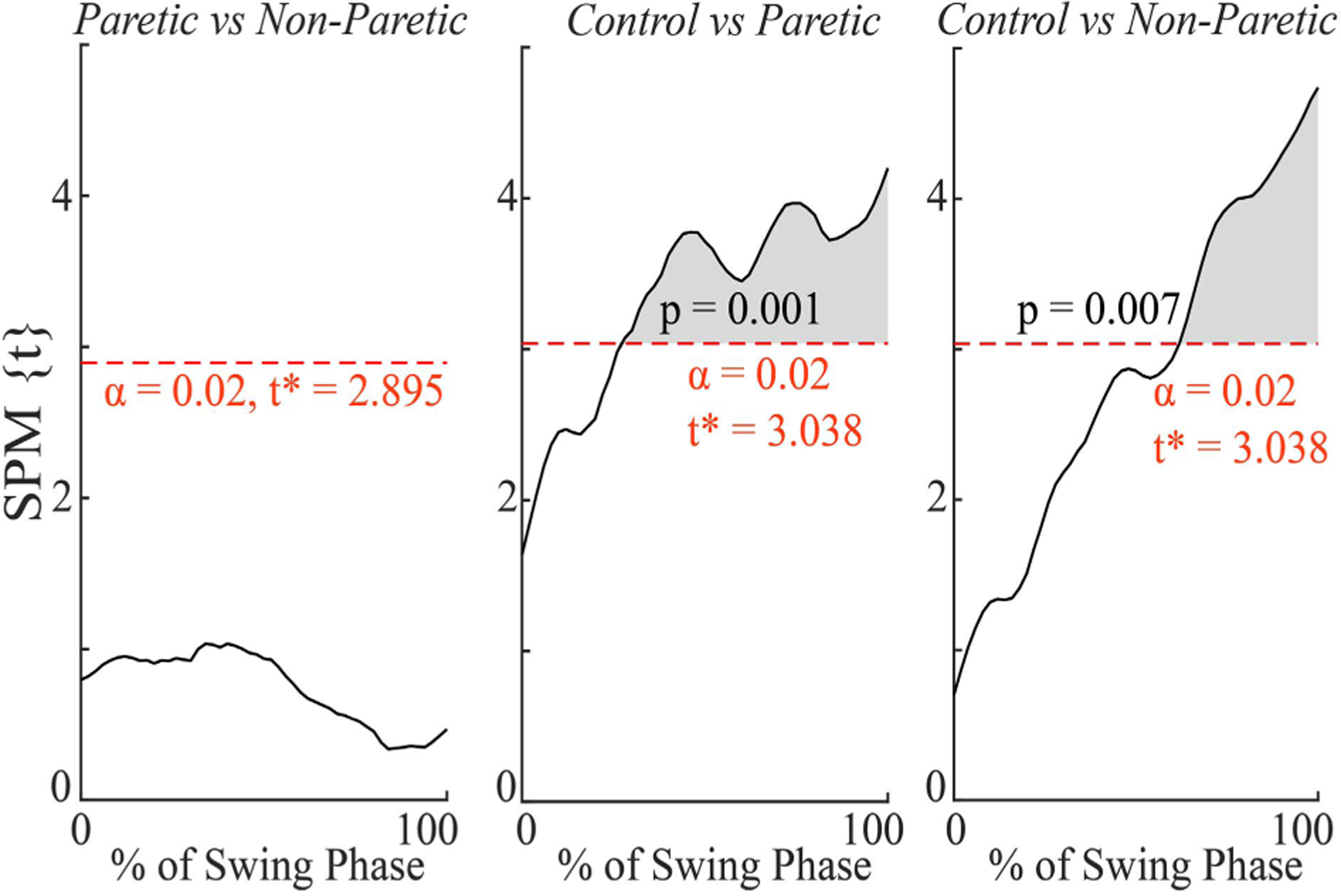
Differences in R2 time-series between limb pairs (Paretic vs Non-Paretic, Control vs Paretic, Control vs Non-Paretic) with Bonferroni corrected p-values. The dashed red line indicates the threshold t-value, corresponding to an *α* = 0.02, where values above this threshold are statistically significant. Shaded regions indicate significant effects for the corresponding portion of the swing phase.

There was a significant main effect of limb (F(1,17) = 4.451, p = 0.01) between 30% – 100% of the swing phase, but not of field or the interaction between the two (Fig. 3). Comparing the R^2^ time-series values between the limbs (Fig. 4), there were significant differences between the paretic and control limbs (Control > Paretic, t(1,29) = 3.038, p = 0.001) from 28%−100% of the swing phase and between the non-paretic and control limbs (Control > Non-Paretic, t(1,29) = 3.038, p = 0.007) from 62% – 100% of the swing phase. There were no significant differences in R^2^ time-series between the paretic and non-paretic limbs.

### D. Gait Metrics

**Fig. 5.**
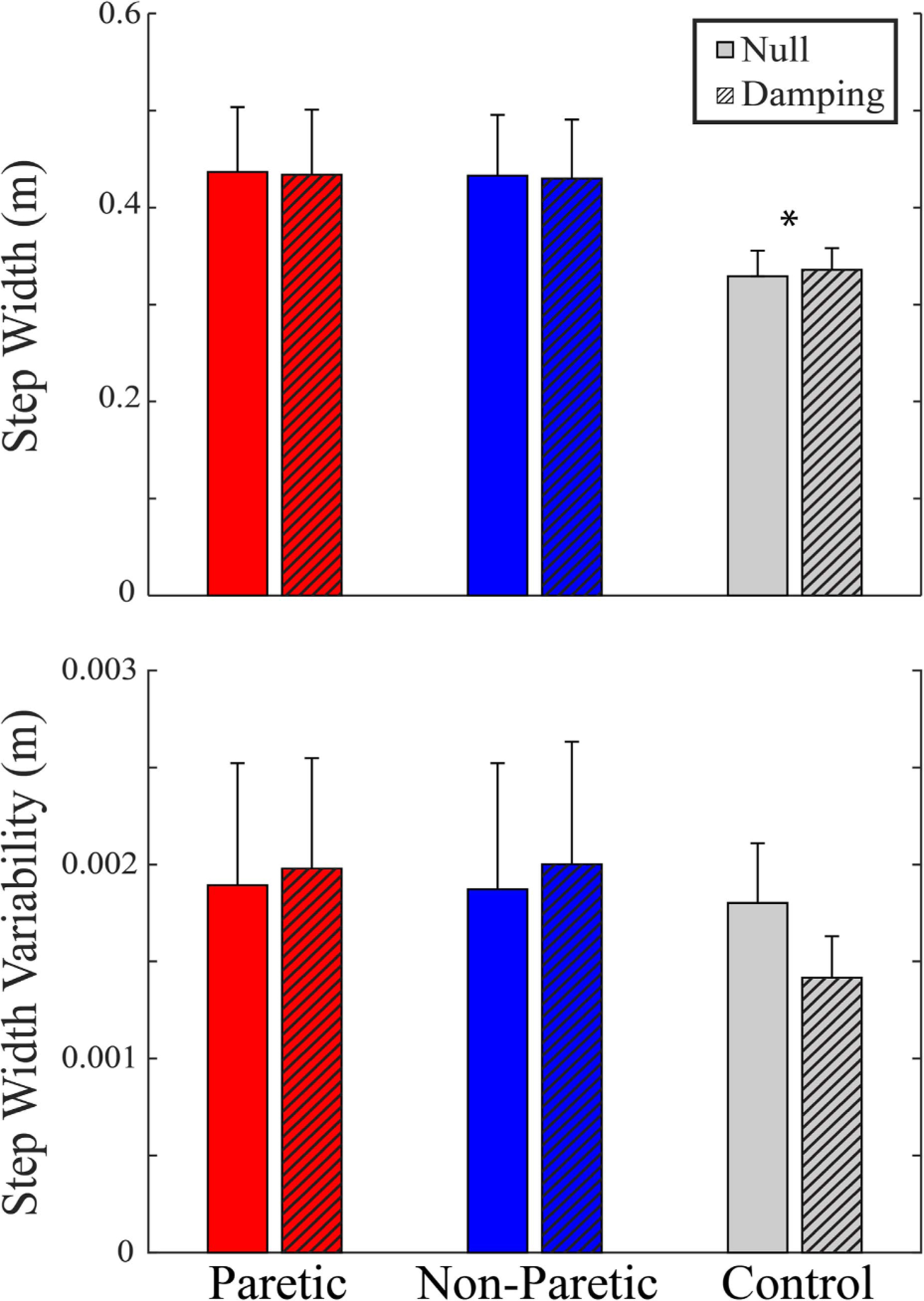
Gait metrics means ± standard errors, Step Width and Step Width Variability. * indicates the Control limb exhibited a signifi-cantly smaller step width than the Paretic or Non-Paretic limbs (Paretic > Control, p = 0.001; Non-Paretic > Control, p = 0.001).

To determine how participants adapted their gait between limbs (Paretic, Non-Paretic, and Control) and within the fields (Null and Damping), we examined the average step width and step width variability (Fig. 5). For step width, there was a significant effect of limb (p = 0.001), but not of field (p = 0.958) or the interaction of limb and field (p = 0.696). Pairwise comparisons found wider steps for the paretic and non-paretic extremities compared to the control extremity (Paretic > Control, p = 0.001; Non-Paretic > Control, p = 0.001). For step width variability, there were no significant effects of limb (p = 0.283), field (p = 0.566), or the interaction of limb and field (p = 0.068).

## IV. Discussion

The purpose of this study was to investigate if individuals with stroke adapt their step-to-step coordination between lateral COM state and lateral foot placement in response to significant changes in lateral stability demands. Here we imposed an external force field that resisted lateral COM velocity to probe the effect of COM state changes during walking on lateral foot placement. We investigated if individuals with and without stroke adapted their stepping strategy (correlation of lateral COM state and lateral foot placement position) and gait parameters (step width mean and variability) in response to a Damping Field. We hypothesized that in comparison to the Null field, the Damping Field would result in a weaker correlation between COM state and foot placement, as well as a reduction in step width mean and variability. This hypothesis was not supported, suggesting that there is a complex relationship between an individual’s stepping strategy and the demands to maintain lateral stability. Additionally, we hypothesized that the correlation between COM state and foot placement would be weaker for individuals with stroke (both paretic and non-paretic extremities) than for individuals without stroke. This second hypothesis was supported and highlights the differences in mechanisms people with and without stroke use to maintain gait stability.

### A. Differences Between Null and Damping Fields

Our hypothesis that correlations between lateral COM state and lateral foot placement would be weaker in the velocity-resistant Damping Field than in a Null Field was not supported. This hypothesis was based on previous research suggesting that the strength of the coupling between lateral COM state and lateral foot placement scale to the requirements to maintain lateral stability. We quantified the effect of the Damping Field on the relationship between lateral COM state and lateral foot placement by comparing a full swing-phase R^2^ time-series between fields, but found no significant effect of field. For the paretic and non-paretic limbs, R^2^ values were greater at foot-off and initial contact in the Damping Field than in the Null Field and consistently higher throughout the swing phase (Fig. 2). These results suggest a complex relationship between an individual’s stepping strategy and the demands to maintain lateral stability.

Some previous experimental and simulation studies have suggested that there is an active control strategy for maintaining lateral stability that adjusts lateral foot placement relative to the COM state. By recruiting frontal plane abductors in the stance [31] and swing limbs [31], [32], an individual can alter their step-to-step lateral foot placement to account for lateral COM state. This strategy is utilized during the early to mid-swing phase when the nervous system can make corrections to lateral foot placement, whereas the latter half of the swing phase is primarily influenced by the passive dynamics of the swing limb. Previous studies in individuals without neurological injury have found that this correlation is adaptable to the demands to maintain lateral stability through the application of external lateral forces, where external lateral stabilization decreases the correlation [4] and lateral movement amplification increases the correlation [18], [20], although these correlation changes may be driven by manipulation of COM dynamics by the forces themselves. We expected the Damping Field to disrupt the coordination between the lateral COM and lateral foot placement; however, the Damping Field did not lead to differences in correlations or step width between the two fields. We suspect that the lack of changes in the Damping Field may be partly explained by a decreased sensitivity to manipulations of lateral COM velocity compared to COM position. To determine the relative sensitivity of the two regression coefficients, we examined the regression coefficients between the two fields (and the three limbs) and found that they were quite similar. We found that *β*_1_ values (COM position) were consistently positive (*β*_1_ ≈ 0.9 at mid-stance) and significant throughout the swing phase for all conditions and limbs. *β*_2_ values (COM velocity) had smaller magnitudes (*β*_2_ ≈ 0.1 at mid-stance) and were non-significant during the swing phase from 20–60% depending upon the condition and limb. The magnitudes of our regression coefficients are smaller than has previously been reported at mid-stance (*β*_1_ ≈ 2.01 and *β*_2_ ≈ 0.44) [1], meaning deviations of the COM position or velocity led to smaller changes in lateral foot placement position than previous reports. For both individuals with and without stroke, COM position explains most of the variability in lateral foot placement position. Our participants were older than those in previous studies and may have experienced age-related changes in COM stability [33] and hip abductor strength [34] that affected the relationship between lateral COM state and lateral foot placement position. Ultimately, this reduced sensitivity to COM velocity deviations may have contributed to the lack of changes seen in the correlations when walking in our velocity-based Damping Field compared to previously-examined position-based fields.

### B. Differences Between Limbs

In support of our second hypothesis, we found individuals with stroke had significantly weaker correlations between lateral COM state and lateral foot placement compared to individuals without stroke across the fields. These differences between the limbs emerged at ~28 and 62% of the swing cycle. In addition, while both groups increased R^2^ values from foot-off to initial contact, the magnitude of this increase was 2–3x greater in the individuals without stroke. These results suggest that the strategies used to coordinate COM state and foot placement are different for individuals with and without stroke. We suspect the minimal changes in correlations from the start to the end of swing phase for the paretic and non-paretic extremities is indicative of a compensatory, non-specific stepping strategy.

If an individual is using a specific strategy to coordinate foot placement, one would expect the correlation between lateral COM state and lateral foot placement to strengthen throughout the swing phase, particularly during the first half. For an individual with an intact neuro-musculoskeletal system, there would be sufficient time to accurately analyze their COM state and make corrections to their swing-limb trajectory with an appropriate foot placement. Our observations of individuals without stroke found that R^2^ values in the Null Field increased from 0.46 at foot-off to 0.76 at initial contact, suggesting that this group may be using this specific control strategy.

If an individual were relying on a non-specific strategy for maintaining lateral stability by selecting an indiscriminately wide step width, COM state would not become increasingly predictive of foot placement during the progression through the swing phase. This strategy may decrease the contribution of passive dynamics on the observed correlations. Other recent work has also suggested similar increases in reliance on non-specific strategies to maintain gait stability in individuals with incomplete spinal cord injury [15], [16] and stroke [6]. Our observations of individuals with stroke were consistent with this non-specific strategy, as participants in the current study took significantly wider steps than their peers without stroke and their R^2^ values only increased by 0.09 or 0.10 from foot-off to initial contact for the paretic and non-paretic extremities respectively when walking in the Null Field. A reliance on non-specific strategies may be beneficial to individuals with stroke, who may have sensory [9] and motor deficits [7], [35] that impair their ability to control lateral foot placement during walking. These deficits include a reduced ability to target and choose an appropriate foot placement [8], [35], ability to regulate prescribed step widths [7], and proprioception of lateral hip musculature [9]. They are further limited by neurologic deficits, such as abnormal lower extremity torque synergies [10], [11], increases in spasticity [12], the adoption of compensatory paretic hip circumduction and non-paretic hip hiking [36], all of which may limit the coordination of lower extremity trajectories and torques. Our results suggest that the coordination of lateral foot placement relative to lateral COM state is significantly different between individuals with and without stroke. Our results differ from previous work that found individuals with stroke behave more similarly to those without stroke [6], although these differences may be due to the heterogeneity of the post-stroke population that possess a wide range of functional abilities.

### C. Limitations

There are two important limitations to consider when interpreting these results. The first is the small sample size (n = 9 in both groups). Depending on the variable of interest, we found statistically significant differences between groups with significance that ranged from p = 0.05 to 0.001; however, these results should be interpreted with caution. Our small sample size limits the practical significance of our results, and future work that investigates the effects of the Damping Field with a larger sample size should be conducted to better distinguish the differences in stepping strategy between individuals with and without stroke.

The second limitation is the differences in average gait speeds between the two groups. Individuals without stroke walked with average gait speeds that were twice as fast as individuals with stroke. Previous research has found that correlations between lateral COM state and lateral foot placement are sensitive to gait speed in individuals with an intact neuro-musculoskeletal system [20], [37]. However, while the group differences in R^2^ in early swing phase may be greatly influenced by speed, the differences in late swing phase may be more significantly affected by differences in stepping strategy and neurologic changes following a stroke. Here we were interested in measuring the differences in stepping strategy at an individual’s preferred walking speed, but further work should investigate how these correlations are influenced by changes in gait speed for individuals with stroke.

### D. Clinical Implications

Individuals with stroke often have difficulty maintaining gait stability, putting them at a higher risk for falling than their peers without neurological injury [38]. Challenges to stability post-stroke can arise from internal factors, such as distal lower extremity weakness [39], spasticity [12], and abnormal torque synergies [10], [11]. These internal factors limit the adaptability of the post-stroke neuromuscular control system to meet the mechanical requirements of gait and contribute to the observed adoption of abnormal gait patterns [40] and compensatory behaviors [36]. It is likely that these internal factors and their requisite compensations may impede the capacity of post-stroke gait to respond to changes in the external environment using stabilization strategies that require coordination between the COM and lower extremities. Our results suggest that in comparison to their peers without stroke, individuals with stroke utilize a more non-specific strategy for maintaining lateral stability. Rather than making step-to-step adjustments of lateral foot placement that are coupled to their ongoing COM dynamics, individuals with stroke demonstrate a compensatory strategy that prioritizes a wider step width. While this strategy may provide a robust solution for maintaining lateral stability, it has trade-offs, including increasing the metabolic energy cost of walking [41] and limiting maneuverability [42]. Interventions that can strengthen the correlation between COM state and lateral foot placement may be beneficial for practicing some desirable locomotor stability strategies. For example, the application of force fields at the COM [20] or at the swing limb [43] can effectively influence the relationship between the COM dynamics and foot placement. For clinical populations that utilize compensatory, non-specific strategies for maintaining lateral stability, these interventions may have the potential to be used to improve coordinated stepping behaviors.

## Figures and Tables

**TABLE I:** Participant Demographics and Clinical Outcome Measures

	Sex	Age	Paretic Side	Preferred Speed (m/s)	10MWT Self	10MWT Fast	BBS	FMA LE
**Stroke**								
S1	M	61	R	0.58	0.38	0.68	55	23
S2	M	58	R	0.40	0.40	0.53	53	17
S3	M	47	L	0.49	0.44	0.67	55	23
S4	F	64	R	0.18	0.31	0.37	50	19
S5	F	68	R	0.36	0.38	0.51	55	19
S6	M	49	R	0.72	0.53	0.76	45	21
S7	M	63	R	0.22	0.22	0.14	32	19
S8	M	64	L	0.36	0.69	0.86	53	23
S9	M	58	L	0.58	0.69	0.93	51	22
*Average*		59		0.43	0.45	0.60	50	21
*STD*		7		0.17	0.15	0.23	7	2
**Control**								
C1	M	64		1.07	1.30	1.70		
C2	M	71		1.03	1.23	2.03		
C3	M	62		0.94	1.30	1.70		
C4	M	56		1.12	1.60	2.13		
C5	M	67		0.67	1.13	1.81		
C6	M	64		0.85	1.10	1.50		
C7	M	56		1.30	1.50	2.20		
C8	M	55		1.21	1.40	1.90		
C9	M	50		1.21	1.50	2.20		
*Average*		61		1.04	1.34	1.91		
*STD*		6		0.19	0.16	0.24		

**TABLE II:** R^2^ Data

	Null Field		Damping Field	
	*Foot-off*	*Initial Contact*	*Foot-off*	*Initial Contact*
**Paretic**	0.35 ± 0.17	0.44 ± 0.25	0.47 ± 0.21	0.54 ± 0.25
**Non-Paretic**	0.40 ± 0.18	0.50 ± 0.18	0.53 ± 0.21	0.54 ± 0.19
**Control**	0.46 ± 0.11	0.76 ± 0.12	0.55 ± 0.15	0.74 ± 0.04
